# Supplementary material for: Experimental nonclassicality in a causal network without assuming freedom of choice
Source: Nat Commun. 2023 Feb 17;14:909. doi: 10.1038/s41467-023-36428-w (PMC9938195; doi:10.1038/s41467-023-36428-w)
Supplement: Supplementary file 1 — Supplementary Information [file 41467_2023_36428_MOESM1_ESM.pdf]

Supplementary Information for Experimental nonclassicality in a causal network without assuming freedom of choice

Emanuele Polino,[1] Davide Poderini,[1] Giovanni Rodari,[1] Iris Agresti,[1] Alessia Suprano,[1] Gonzalo Carvacho,[1] Elie Wolfe,[2, *] Askery Canabarro,[3, 4] George Moreno,[3, 4] Giorgio Milani,[1] Robert W. Spekkens,[2] Rafael Chaves,[5, †] and Fabio Sciarrino[1, ‡]

[1] *Dipartimento di Fisica - Sapienza Università di Roma, P.le Aldo Moro 5, I-00185 Roma, Italy*
[2] *Perimeter Institute for Theoretical Physics, 31 Caroline St. N, Waterloo, Ontario, N2L 2Y5, Canada*
[3] *International Institute of Physics, Federal University of Rio Grande do Norte, 59078-970, Natal, RN, Brazil*
[4] *Grupo de Física da Matéria Condensada, Núcleo de Ciências Exatas - NCEx, Campus Arapiraca, Universidade Federal de Alagoas, 57309-005, Arapiraca, AL, Brazil*
[5] *International Institute of Physics & School of Science and Technology, Federal University of Rio Grande do Norte, 59078-970, P. O. Box 1613, Natal, Brazil*

* ewolfe@perimeterinstitute.ca
† rchaves@iip.ufrn.br
‡ fabio.sciarrino@uniroma1.it

Supplementary Note 1. DATA ANALYSIS

The data acquisition was performed using three independent time-to-digital converters (TDC) with a resolution of $\approx$81 ps. The TDCs, one for each measurement station, were synchronized using a shared random signal, acting as time reference. The synchronization was performed in real time, via dedicated software running in a separated machine which received data from the three nodes on the local network. After the synchronization, the data coming from the TDCs are filtered to retain only the events corresponding to a two-fold coincidence detection between events coming from the same source. Such events are then saved to disk for further analysis and post processing. The time window in which two events are considered coincident is $\approx$4.1 ns, this allows to filter out most noise sources affecting the measurement. The array of two-fold coincidences obtained is further analyzed to count coincidence events among all the three parties. A six-fold event extracted in this way is a set of three two-fold events that can be considered coincident in a given window, larger than the one used to extract two-fold coincidences. When more than three two-fold coincidences are found in the same time window, the additional events are discarded. More specifically, when more than one coincidence from a same source are found within the window interval, we select only the first detected. Selecting the first coincidence represents an arbitrary and unbiased choice, that is a useful convention for resolving possible ambiguities in the definition of six-fold events.

Supplementary Note 2. EXPERIMENTAL FRITZ DISTRIBUTION

We show the experimental frequencies corresponding to the terms of Fritz distribution in Supp. Fig. 1-a, in comparison with the ideal values (Supp. Fig. 1-b). The overall statistics is composed of $\sim 10^6$ events.

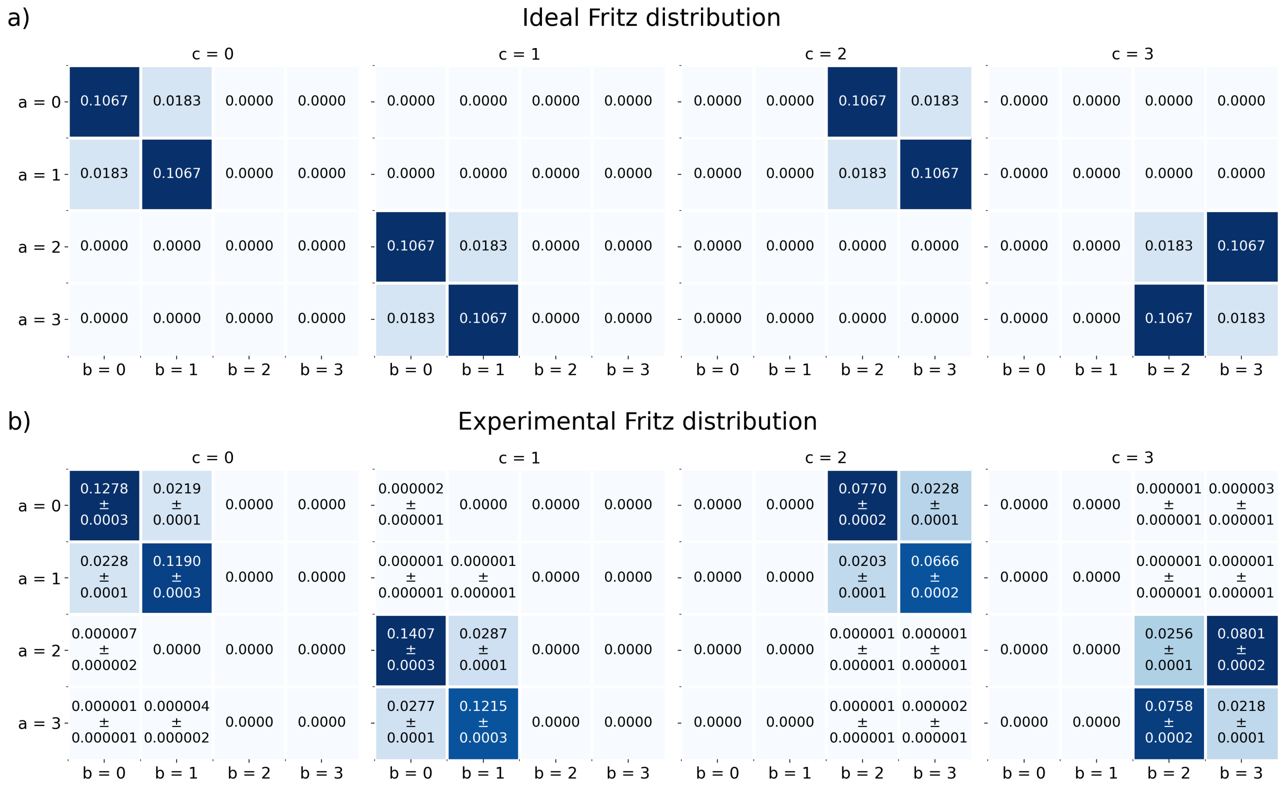

x

Supplementary Figure 1. **Experimental Fritz distribution. a)** Theoretical Fritz distribution considering ideal noiseless singlet states. **b)** Experimental distribution measured in an experimental run.

Supplementary Note 3. NEURAL NETWORK ORACLES

Given a causal structure and a distribution over the observed outputs, we used an ensemble of neural networks to determine whether the distribution could have been reproduced by using exclusively local resources. The general idea is to encode the causal structure into neural networks and request them to reproduce the target distribution. Both the assumed causal structures and a feedforward neural networks can have their information flow determined by a directed acyclic graph (DAG). Therefore, we train diverse neural networks taking into consideration the causal structure to reproduce the target distribution.

Consider the scenario in which three sources $(\lambda_{AB},\lambda_{AC},\lambda_{BC})$, send information either via a classical or quantum channel to three parties, Alice (A), Bob (B), and Charlie (C) with the following constrained flow of information: each source only sends information to two parties of the three, constituting a triangle network with quaternary (four possible flag numbers) outputs and no inputs, as described in the main text. In this manner, Alice, Bob and Charlie receives, respectively, the pairs $\lambda_A = \{\lambda_{AB},\lambda_{AC}\}$, $\lambda_B = \{\lambda_{AB},\lambda_{BC}\}$ and $\lambda_C = \{\lambda_{AC},\lambda_{BC}\}$. Therefore, individual inputs $\in \mathbb{R}^2$ (they have length 2). For training, we provide batches of $(N_{\text{batch}},2)$ dimension for the corresponding MLP for each party.

The parties process their inputs by means of arbitrary local response functions, characterized by the conditional probabilities $p(a|\lambda_{AB},\lambda_{AC})$, $p(b|\lambda_{AB},\lambda_{BC})$ and $p(c|\lambda_{AC},\lambda_{BC})$, where $a,b,c \in \{0,1,2,3\}$ are the flag numbers by the parties A,B,C, respectively. Although any other distribution can be reabsorbed by the established parties' response functions $p_X(x|\lambda_{xy},\lambda_{xz})$, for the classical setup one can assume that the sources send a random variable taken from an uniform distribution in the unit interval, i.e. $\lambda_{AB},\lambda_{BC},\lambda_{AC} \in [0,1]$.

Therefore, given these constraints and the assumption that each source is independent, such a scenario is well characterized by the probability distribution $p(a,b,c)$ over the random variables of the outputs [1], which be written as:

$$p(a,b,c) = \iiint_0^1 p(a|\lambda_{AB},\lambda_{AC})p(b|\lambda_{AB},\lambda_{BC})p(c|\lambda_{AC},\lambda_{BC})d\lambda_{AB}d\lambda_{AC}d\lambda_{BC}\,. \tag{1}$$

The aim in the machine learning (ML) part of this work is to construct neural networks capable of approximating distributions given by Eq. 1. Amid the myriad of ML algorithms that exist nowadays, feedforward neural networks are the one to emulate a directed acyclic graph (DAG) corresponding to a causal structure. This symmetry between causal structure and neural network is a powerful trait making the method applicable, *a priori*, to any causal structure [1].

For each party (A,B,C), the corresponding response function will be incorporated by means of a fully connected multilayer perceptron (MLP). However, in principle, more advanced neural networks architectures could be used as well, such as convolutional neural networks (CNNs), recurrent neural networks (RNNs) and so on, but this would require a dedicated investigation that would be far beyond the scope of this paper, although the implementation of an ensemble of MLPs is already a natural advance of the inaugural method shown in Ref. [1].

From the machine learning perspective, the input layers to the MLPs are composed of the independent uniformly distributed random numbers in the unit interval, i.e. $\lambda_{AB},\lambda_{BC},\lambda_{AC} \in [0,1]$, with the restriction in the flow of information imposed by the triangle network: each source only sends information to two parties of the three. In this manner, Alice receives $(\lambda_{AB},\lambda_{AC})$, Bob receives $(\lambda_{AB},\lambda_{BC})$ and Charlie receives $(\lambda_{BC},\lambda_{AC})$. See the diagram in Supp. Fig. 2. Therefore, individual inputs $\in \mathbb{R}^2$ (they have length 2). For training, we provide batches of $(N_{\text{batch}},2)$ dimension for the corresponding MLP for each party. The output layers retrieve the corresponding probabilities conditioned on the respective inputs: $p(a|\lambda_{AB},\lambda_{AC})$, $p(b|\lambda_{AB},\lambda_{BC})$ and $p(c|\lambda_{AC},\lambda_{BC})$. In this step, we use a softmax activation function so that the outputs are three normalized vectors $\in \mathbb{R}^4$ (length 4).

We then evaluate the neural network for N_{batch} sample values of random variables $(\lambda_{AB},\lambda_{AC},\lambda_{BC})$ in order to approximate the joint probability distribution Eq. 1, averaging the Cartesian product of the conditional probabilities ($\in \mathbb{R}^4$),

$$\tilde{p}(a,b,c) = \frac{1}{N_{\text{batch}}}\sum_{i=1}^{N_{\text{batch}}} p(a|\lambda_{AB\,i},\lambda_{AC\,i})p(b|\lambda_{AB\,i},\lambda_{BC\,i})p(c|\lambda_{AC\,i},\lambda_{BC\,i}), \tag{2}$$

yielding the approximated joint probability distribution $\tilde{p}(a,b,c) \in \mathbb{R}^{64}$ with the following index ordering: $[0,1,2,3]\times[0,1,2,3]\times[0,1,2,3] \to [000,001,002,...,333]$. In this manner, the method is order sensitive. This step formally differs from the Monte Carlo approximation in Ref. [1], however, in practice, they yield the same result for the examples we tested.

The approximation of $p(a,b,c)$ is achieved by setting a loss function quantifying the discrepancy between the target distribution $p(a,b,c)$ and the neural network's constructed output $\tilde{p}(a,b,c)$. A natural candidate is therefore the relative Kullback–Leibler divergence,

$$KL(p,\tilde{p}) = \sum_{abc} p(a,b,c)\log\left(\frac{p(a,b,c)}{\tilde{p}(a,b,c)}\right). \tag{3}$$

In fact, any differentiable discrepancy measure between p and $\tilde{p}$ works (for instance, the MSE (l2-norm), the MAE (l1-norm), Euclidean distance, and so on). However, the KL seems to be the best on the practical side (faster and better convergence), a point also registered in Ref. [1].

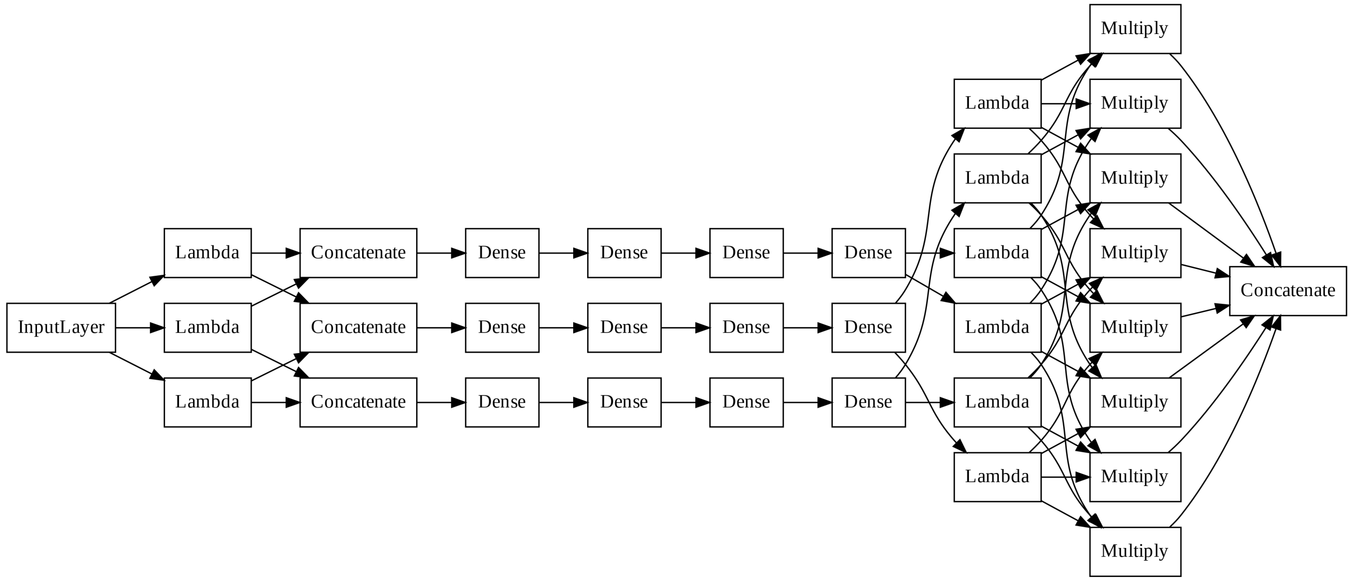

Supplementary Figure 2. **ML diagram.** Scheme of the NN architecture.

As already mentioned, for an individual neural network forming the committee, we used a similar approach as in [1]. The ensemble of neural networks, which can be imagined as an assembly of oracles, provides a certificate that a distribution is local once it is learned. In other words, if a target distribution is inside the local set, then a sufficiently expressive neural network should be able to learn the appropriate response functions and reproduce it. As the "right" expressivity is not known *a priori*, we decided to use the ensemble approach, in which we train diverse neural networks with distinct number of layers and neurons. For distributions outside the local set, we should see that the machine can not approximate well the given target, no matter the expressitivy power we employ for the neural network. So, the ensemble is a good indication (not a definite proof) of this impossibility. Altogether, this gives us a criterion for deciding whether a target distribution is inside the local set or not.

Heuristically, it means that, by construction, the neural network can learn the local responses of the parties to their inputs. Notwithstanding, if a given target is surely outside the local set (quantum channels are permitted from the sources to the parties), then by adding noise according to a convex sum we should see a clear transition in function of visibility in the learner's behavior when entering the set of local correlations, as demonstrated in the main text. The distance between the target and learned distributions can be computed in a number of ways. However, the element-wise mean square error (MSE) also known as l2-norm error between $p(a,b,c)$ and $\tilde{p}(a,b,c)$, is a better guide to the eyes for identifying the transition.

The advantage of using multiple neural networks architectures is well perceived by examining Supp. Tab. 1. Note that for distinct visibility values, a different neural network architecture yields the best MSE distance, strengthening the advantage of using an assembly of oracles.

Supplementary Table 1. **Optimal NN parameters.**The corresponding best architecture yielding the minimum MSE distance for each visibility value v.

Visibility (v)	Optimum Architecture
0.00	5 layers, 16 neurons
0.05	4 layers, 16 neurons
0.10	5 layers, 16 neurons
0.15	4 layers, 16 neurons
0.20	5 layers, 32 neurons
0.25	4 layers, 32 neurons
0.30	6 layers, 32 neurons
0.35	4 layers, 32 neurons
0.40	3 layers, 32 neurons
0.45	3 layers, 32 neurons
0.50	3 layers, 32 neurons
0.55	6 layers, 32 neurons
0.60	3 layers, 16 neurons
0.65	5 layers, 16 neurons
0.70	5 layers, 32 neurons
0.75	6 layers, 16 neurons
0.80	3 layers, 32 neurons
0.85	3 layers, 32 neurons
0.90	5 layers, 16 neurons
0.95	3 layers, 32 neurons
1.00	5 layers, 16 neurons

Supplementary Note 4. CAUSAL COMPATIBILITY INEQUALITY FROM THE INFLATION TECHNIQUE

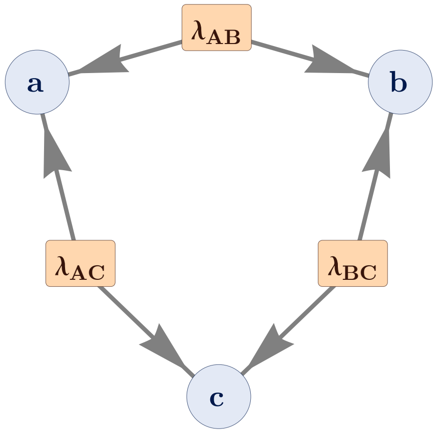

Supplementary Figure 3. **Triangle DAG.** The causal structure of the classical triangle network, a falsified hypothesis for explaining our experimental statistics.

Here's how the second order inflation test works for the triangle network. Suppose that a distribution $p(a,b,c)$ is compatible with the triangle causal structure in Supp. Fig. 3, in the sense of

$$p(abc)=\sum_{\lambda_{AB}\lambda_{AC}\lambda_{BC}} p(a|\lambda_{AB},\lambda_{AC})p(b|\lambda_{AB},\lambda_{BC})p(c|\lambda_{AC},\lambda_{BC})p(\lambda_{AB})p(\lambda_{AC})p(\lambda_{BC}). \tag{4}$$

Now, imagine (gedankenexperiment) recycling the same causal components to create some hypothetically observable distribution $p'(a^{(1)},b^{(1)},c^{(1)},a^{(2)},b^{(2)},c^{(2)},a^{(3)},b^{(3)},c^{(3)},a^{(4)},b^{(4)},c^{(4)})$ compatible with the second order inflation graph of the triangle scenario, depicted in Fig. 4. Relative to the inflation graph, compatibility means

$$p'(a^{(1)},b^{(1)},c^{(1)},a^{(2)},b^{(2)},c^{(2)},a^{(3)},b^{(3)},c^{(3)},a^{(4)},b^{(4)},c^{(4)})=\sum_{\substack{\lambda_{AB}^{(1)},\lambda_{AB}^{(2)},\\ \lambda_{AC}^{(1)},\lambda_{AC}^{(2)},\\ \lambda_{BC}^{(1)},\lambda_{BC}^{(2)}}} \begin{pmatrix} p'(a^{(1)}|\lambda_{AB}^{(1)},\lambda_{AC}^{(1)})p'(b^{(1)}|\lambda_{AB}^{(1)},\lambda_{BC}^{(1)})p'(c^{(1)}|\lambda_{AC}^{(1)},\lambda_{BC}^{(1)}) \\ \times p'(a^{(2)}|\lambda_{AB}^{(2)},\lambda_{AC}^{(1)})p'(b^{(2)}|\lambda_{AB}^{(1)},\lambda_{BC}^{(2)})p'(c^{(2)}|\lambda_{AC}^{(2)},\lambda_{BC}^{(1)}) \\ \times p'(a^{(3)}|\lambda_{AB}^{(1)},\lambda_{AC}^{(2)})p'(b^{(3)}|\lambda_{AB}^{(2)},\lambda_{BC}^{(1)})p'(c^{(3)}|\lambda_{AC}^{(1)},\lambda_{BC}^{(2)}) \\ \times p'(a^{(4)}|\lambda_{AB}^{(2)},\lambda_{AC}^{(2)})p'(b^{(4)}|\lambda_{AB}^{(2)},\lambda_{BC}^{(2)})p'(c^{(4)}|\lambda_{AC}^{(2)},\lambda_{BC}^{(2)}) \\ \times p'(\lambda_{AB}^{(1)})p'(\lambda_{AB}^{(2)})p'(\lambda_{AC}^{(1)})p'(\lambda_{AC}^{(2)})p'(\lambda_{BC}^{(1)})p'(\lambda_{BC}^{(2)}) \end{pmatrix} \tag{5}$$

By "recycling causal components" we mean that the functional dependence of every variable on its parents in Supp. Fig. 4 is presumed to be the same functional dependence as the analogous variable's functional dependence on its parents in Supp. Fig. 3. The Supp. Fig. 3 analog of each individual variable in Supp. Fig. 4 is obtained by dropping the superscript copy index. Hereafter, when describing random variables being assigned particular values we place the name of the variable in underscript and the value it takes in overscript.

That is,

$$\begin{aligned} & p'(\underset{a^{(1)}|\lambda_{AB}^{(1)},\lambda_{AC}^{(1)}}{v|\mu_1,\mu_2})=p'(\underset{a^{(2)}|\lambda_{AB}^{(2)},\lambda_{AC}^{(1)}}{v|\mu_1,\mu_2})=p'(\underset{a^{(3)}|\lambda_{AB}^{(1)},\lambda_{AC}^{(2)}}{v|\mu_1,\mu_2})=p'(\underset{a^{(4)}|\lambda_{AB}^{(2)},\lambda_{AC}^{(2)}}{v|\mu_1,\mu_2})=p(\underset{a|\lambda_{AB},\lambda_{AC}}{v|\mu_1,\mu_2}), \\ \text{and}\quad & p'(\underset{b^{(1)}|\lambda_{AB}^{(1)},\lambda_{BC}^{(1)}}{v|\mu_1,\mu_2})=p'(\underset{b^{(2)}|\lambda_{AB}^{(1)},\lambda_{BC}^{(2)}}{v|\mu_1,\mu_2})=p'(\underset{b^{(3)}|\lambda_{AB}^{(2)},\lambda_{BC}^{(1)}}{v|\mu_1,\mu_2})=p'(\underset{b^{(4)}|\lambda_{AB}^{(2)},\lambda_{BC}^{(2)}}{v|\mu_1,\mu_2})=p(\underset{b|\lambda_{AB},\lambda_{BC}}{v|\mu_1,\mu_2}), \\ \text{and}\quad & p'(\underset{c^{(1)}|\lambda_{AC}^{(1)},\lambda_{BC}^{(1)}}{v|\mu_1,\mu_2})=p'(\underset{c^{(2)}|\lambda_{AC}^{(2)},\lambda_{BC}^{(1)}}{v|\mu_1,\mu_2})=p'(\underset{c^{(3)}|\lambda_{AC}^{(1)},\lambda_{BC}^{(2)}}{v|\mu_1,\mu_2})=p'(\underset{c^{(4)}|\lambda_{AC}^{(2)},\lambda_{BC}^{(2)}}{v|\mu_1,\mu_2})=p(\underset{c|\lambda_{AC},\lambda_{BC}}{v|\mu_1,\mu_2}), \\ \text{and}\quad & p'(\underset{\lambda_{AB}^{(1)}}{\mu})=p'(\underset{\lambda_{AB}^{(2)}}{\mu})=p(\underset{\lambda_{AB}}{\mu}), \quad \text{and}\quad p'(\underset{\lambda_{AC}^{(1)}}{\mu})=p'(\underset{\lambda_{AC}^{(2)}}{\mu})=p(\underset{\lambda_{AC}}{\mu}), \quad \text{and}\quad p'(\underset{\lambda_{BC}^{(1)}}{\mu})=p'(\underset{\lambda_{BC}^{(2)}}{\mu})=p(\underset{\lambda_{BC}}{\mu}). \end{aligned} \tag{6}$$

Now, Eq. (6) holds whenever $p(abc)$ is compatible with the triangle causal structure. Naturally, in our case, Eq. (6)

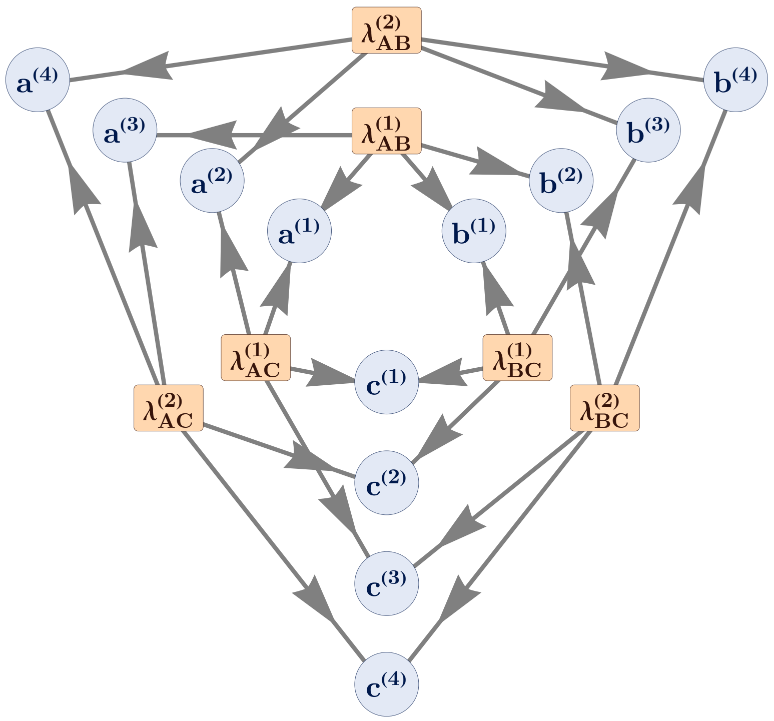

Supplementary Figure 4. **Triangle inflation DAG.** The second order inflation graph of the triangle network.

by itself is not operationally testable. We only have access to the observed distribution $p(abc)$; if we had access to the underlying functional dependencies such as $p(a|\lambda_{AB},\lambda_{AC})$ we wouldn't be bother with inflation, as we would then be able to access the (in)compatibility of $p(abc)$ by means of Eq. (4) alone. The key insight is to extract easily testable implications of Eq. (6) which follow merely from the (non)existence of suitable functional dependencies. The two implications of Eq. (6) which we elect to incorporate into our linear program feasibility test are **marginalization** and **symmetry**.

The marginalization condition is that

$$\begin{aligned} p'(\underset{a^{(1)}}{v_1},\underset{b^{(1)}}{v_2},\underset{c^{(1)}}{v_3},\underset{a^{(4)}}{v_{10}},\underset{b^{(4)}}{v_{11}},\underset{c^{(4)}}{v_{12}}) &\coloneqq \sum_{\substack{v_4,v_5,v_6,\\ v_7,v_8,v_9}} p'(\underset{a^{(1)}}{v_1},\underset{b^{(1)}}{v_2},\underset{c^{(1)}}{v_3},\underset{a^{(2)}}{v_4},\underset{b^{(2)}}{v_5},\underset{c^{(2)}}{v_6},\underset{a^{(3)}}{v_7},\underset{b^{(3)}}{v_8},\underset{c^{(3)}}{v_9},\underset{a^{(4)}}{v_{10}},\underset{b^{(4)}}{v_{11}},\underset{c^{(4)}}{v_{12}}) \\ &= p(\underset{a}{v_1},\underset{b}{v_2},\underset{c}{v_3})\,p(\underset{a}{v_{10}},\underset{b}{v_{11}},\underset{c}{v_{12}}), \\ \text{which we express as} \quad &\boldsymbol{M}\cdot p' = p^{\otimes 2}, \end{aligned} \tag{7}$$

where $\boldsymbol{M}$ is a zero/one valued marginalization matrix; it encodes the appropriate linear combinations of probabilities of p' to make up the marginal probabilities. Accordingly, Eq. (7) is a matrix equality constraint, or 4^6 different equality constraints corresponding to the 4^6 rows of $\boldsymbol{M}$, as the cardinality of all observable variables in our triangle network is 4.

The symmetry condition follows from the fact that the copy indices are dummy indices. That is, since $p'(\underset{\lambda^{(1)}_{AB}}{\mu}) = p'(\underset{\lambda^{(2)}_{AB}}{\mu})$ and so on, it follows that p' must be invariant under the relabellings

π_1	π_2	π_3
$\lambda^{(1)}_{AB} \leftrightarrow \lambda^{(2)}_{AB}$	$\lambda^{(1)}_{AC} \leftrightarrow \lambda^{(2)}_{AC}$	$\lambda^{(1)}_{BC} \leftrightarrow \lambda^{(2)}_{BC}$
$a^{(1)} \leftrightarrow a^{(2)}$	$a^{(1)} \leftrightarrow a^{(3)}$	$b^{(1)} \leftrightarrow b^{(2)}$
$a^{(3)} \leftrightarrow a^{(4)}$	$a^{(2)} \leftrightarrow a^{(4)}$	$b^{(3)} \leftrightarrow b^{(4)}$
$b^{(1)} \leftrightarrow b^{(3)}$	$c^{(1)} \leftrightarrow c^{(2)}$	$c^{(1)} \leftrightarrow c^{(3)}$
$b^{(3)} \leftrightarrow b^{(4)}$	$c^{(3)} \leftrightarrow c^{(4)}$	$c^{(2)} \leftrightarrow c^{(4)}$

(8)

which generates an order-8 symmetry group. For maximum clarity, the cumulative effect of the symmetry implications of

Eq. (6) are

$$
\begin{aligned}
&p'(\underset{a^{(1)}}{v_1},\underset{b^{(1)}}{v_2},\underset{c^{(1)}}{v_3},\underset{a^{(2)}}{v_4},\underset{b^{(2)}}{v_5},\underset{c^{(2)}}{v_6},\underset{a^{(3)}}{v_7},\underset{b^{(3)}}{v_8},\underset{c^{(3)}}{v_9},\underset{a^{(4)}}{v_{10}},\underset{b^{(4)}}{v_{11}},\underset{c^{(4)}}{v_{12}})\\
=&p'(\underset{a^{(1)}}{v_1},\underset{b^{(1)}}{v_2},\underset{c^{(1)}}{v_3},\underset{a^{(2)}}{v_4},\underset{b^{(2)}}{v_6},\underset{c^{(2)}}{v_5},\underset{a^{(3)}}{v_8},\underset{b^{(3)}}{v_7},\underset{c^{(3)}}{v_{11}},\underset{a^{(4)}}{v_{12}},\underset{b^{(4)}}{v_9},\underset{c^{(4)}}{v_{10}})\\
=&p'(\underset{a^{(1)}}{v_2},\underset{b^{(1)}}{v_1},\underset{c^{(1)}}{v_4},\underset{a^{(2)}}{v_3},\underset{b^{(2)}}{v_7},\underset{c^{(2)}}{v_8},\underset{a^{(3)}}{v_5},\underset{b^{(3)}}{v_6},\underset{c^{(3)}}{v_9},\underset{a^{(4)}}{v_{10}},\underset{b^{(4)}}{v_{11}},\underset{c^{(4)}}{v_{12}})\\
=&p'(\underset{a^{(1)}}{v_2},\underset{b^{(1)}}{v_1},\underset{c^{(1)}}{v_4},\underset{a^{(2)}}{v_3},\underset{b^{(2)}}{v_8},\underset{c^{(2)}}{v_7},\underset{a^{(3)}}{v_6},\underset{b^{(3)}}{v_5},\underset{c^{(3)}}{v_{11}},\underset{a^{(4)}}{v_{12}},\underset{b^{(4)}}{v_9},\underset{c^{(4)}}{v_{10}})\\
=&p'(\underset{a^{(1)}}{v_3},\underset{b^{(1)}}{v_4},\underset{c^{(1)}}{v_1},\underset{a^{(2)}}{v_2},\underset{b^{(2)}}{v_5},\underset{c^{(2)}}{v_6},\underset{a^{(3)}}{v_7},\underset{b^{(3)}}{v_8},\underset{c^{(3)}}{v_{10}},\underset{a^{(4)}}{v_9},\underset{b^{(4)}}{v_{12}},\underset{c^{(4)}}{v_{11}})\\
=&p'(\underset{a^{(1)}}{v_3},\underset{b^{(1)}}{v_4},\underset{c^{(1)}}{v_1},\underset{a^{(2)}}{v_2},\underset{b^{(2)}}{v_6},\underset{c^{(2)}}{v_5},\underset{a^{(3)}}{v_8},\underset{b^{(3)}}{v_7},\underset{c^{(3)}}{v_{12}},\underset{a^{(4)}}{v_{11}},\underset{b^{(4)}}{v_{10}},\underset{c^{(4)}}{v_9})\\
=&p'(\underset{a^{(1)}}{v_4},\underset{b^{(1)}}{v_3},\underset{c^{(1)}}{v_2},\underset{a^{(2)}}{v_1},\underset{b^{(2)}}{v_7},\underset{c^{(2)}}{v_8},\underset{a^{(3)}}{v_5},\underset{b^{(3)}}{v_6},\underset{c^{(3)}}{v_{10}},\underset{a^{(4)}}{v_9},\underset{b^{(4)}}{v_{12}},\underset{c^{(4)}}{v_{11}})\\
=&p'(\underset{a^{(1)}}{v_4},\underset{b^{(1)}}{v_3},\underset{c^{(1)}}{v_2},\underset{a^{(2)}}{v_1},\underset{b^{(2)}}{v_8},\underset{c^{(2)}}{v_7},\underset{a^{(3)}}{v_6},\underset{b^{(3)}}{v_5},\underset{c^{(3)}}{v_{12}},\underset{a^{(4)}}{v_{11}},\underset{b^{(4)}}{v_{10}},\underset{c^{(4)}}{v_9}).
\end{aligned}
\tag{9}
$$

As a linear program, we essentially have

$$\exists_{p'|p'\geq\mathbf{0}}: \quad \boldsymbol{M}\cdot p' = p^{\otimes 2} \quad \text{and} \quad G_{\boldsymbol{\pi}}\circ p' = p', \tag{10}$$

where $G_{\boldsymbol{\pi}}\circ$ is the group twirling operation, i.e. the projection onto the symmetric subspace of the group.

We can simplify the linear program (10) by replacing the invariance of p' under symmetry with the statement that $p^{\otimes 2}$ is recovered by marginalizing the twirled version of p', that is, $\boldsymbol{M}\cdot(G_{\boldsymbol{\pi}}\circ p') = p^{\otimes 2}$. For that matter, we can just as well apply the left action of the twirling operator on the marginalization matrix, i.e.

$$\left(G_{\boldsymbol{\pi}}^{-1}\circ\boldsymbol{M}\right)\cdot p' := \left(G_{\boldsymbol{\pi}}\circ\boldsymbol{M}^T\right)^T\cdot p' = \boldsymbol{M}\cdot(G_{\boldsymbol{\pi}}\circ p') \tag{11}$$

and hence we can equivalently express the linear program (10) as

$$\exists_{p'|p'\geq\mathbf{0}}: \quad \left(G_{\boldsymbol{\pi}}^{-1}\circ\boldsymbol{M}\right)\cdot p' = p^{\otimes 2} \tag{12}$$

which has the advantage of being easily dualized, i.e. the dual of LP (12) is

$$\nexists_{\boldsymbol{y}|\boldsymbol{y}\cdot\left(G_{\boldsymbol{\pi}}^{-1}\circ\boldsymbol{M}\right)\geq\mathbf{0}}: \quad \boldsymbol{y}\cdot p^{\otimes 2} < 0. \tag{13}$$

Indeed, the existence of a $\boldsymbol{y}$ vector which yields both $\left(G_{\boldsymbol{\pi}}^{-1}\circ\boldsymbol{M}\right)\cdot\boldsymbol{y}\geq\mathbf{0}$ and $\boldsymbol{y}\cdot p^{\otimes 2}<0$ constitutes a certificate of infeasibility for LP (12) by Farkas' duality lemma [2]. Accordingly, we discover the polynomial inequality which witnesses the incompatibility of p with the classical triangle network by explicitly minimizing $\boldsymbol{y}\cdot p^{\otimes 2}$ subject to the constraints $\left(G_{\boldsymbol{\pi}}^{-1}\circ\boldsymbol{M}\right)\cdot\boldsymbol{y}\geq\mathbf{0}$.

In Supp. Tab. 2 we show the coefficients $\boldsymbol{y}$ pertaining to the products of probabilities generating the causal compatibility inequality . The coefficient is zero unless explicitly specified otherwise in Supp. Tab. 2.

Supplementary Table 2. **Tabular representation of the causal inequality.** This table specified the coefficients relative to the pairs of multiplied probabilities that give rise to the polynomial causal inequality. All unspecified coefficients are taken to be zero.

−1→	202021	202131	233000	233110	312021	312131	323000	323110					
	023010	031002	031012	033010	100023	100033	102031	112031	121002	121012	121102	121112	123010
	123100	133010	133100	203010	203100	212001	212011	212101	212111	213010	213100	221212	222031
2→	222121	223020	223030	223120	223130	223200	223210	231212	232031	232121	300223	302001	302011
	302101	302111	302221	302231	303010	303100	310223	313010	313100	321212	321302	322031	322121
	331212	331302	332031	332121	333020	333030	333120	333130	333200	333210	333300	333310	
3→	212031	212121	223010	223100	302031	302121	333010	333100					
	002001	003000	010003	011002	012001	012011	013000	013010	020003	020013	022001	022011	022021
	023020	030003	030013	030023	031022	032001	032011	032021	032031	033020	033030	100003	100013
	101002	101012	101022	101032	102001	102011	102101	103000	103010	103020	103030	103100	110003
	110013	110103	111002	111012	111022	111032	111102	112001	112011	112101	112111	113000	113010
	113020	113030	113100	113110	120003	120013	120023	120033	120103	120113	121022	121032	122001
	122011	122021	122031	122101	122111	122121	123020	123030	123120	130003	130013	130023	130033
	130103	130113	130123	131022	131032	131122	132001	132011	132021	132031	132101	132111	132121
	132131	133020	133030	133120	133130	200003	200013	200020	200021	200022	200023	200030	200031
	200032	200033	200103	200113	200120	200121	200122	200123	200130	200131	200132	200133	201002
	201012	201020	201021	201022	201023	201030	201031	201032	201033	201102	201112	201120	201121
	201122	201123	201130	201131	201132	201133	202020	202022	202023	202030	202031	202032	202033
	202120	202121	202122	202123	202130	202132	202133	202201	203020	203021	203022	203023	203030
	203031	203032	203033	203120	203121	203122	203123	203130	203131	203132	203133	203200	210003
	210013	210020	210021	210022	210023	210030	210031	210032	210033	210103	210113	210120	210121
	210122	210123	210130	210131	210132	210133	210203	211002	211012	211020	211021	211022	211023
	211030	211031	211032	211033	211102	211112	211120	211121	211122	211123	211130	211131	211132
	211133	211202	212020	212021	212022	212023	212030	212032	212033	212120	212122	212123	212130
	212131	212132	212133	212201	212211	213020	213021	213022	213023	213030	213031	213032	213033
	213120	213121	213122	213123	213130	213131	213132	213133	213200	213210	220000	220001	220002
	220003	220010	220011	220012	220013	220023	220033	220100	220101	220102	220103	220110	220111
1→	220112	220113	220123	220133	220203	220213	221000	221001	221002	221003	221010	221011	221012
	221013	221022	221032	221100	221101	221102	221103	221110	221111	221112	221113	221122	221132
	222000	222001	222002	222003	222010	222011	222012	222013	222100	222101	222102	222103	222110
	222111	222112	222113	222201	222211	222221	223000	223001	223002	223003	223011	223012	223013
	223101	223102	223103	223110	223111	223112	223113	223220	230000	230001	230002	230003	230010
	230011	230012	230013	230023	230033	230100	230101	230102	230103	230110	230111	230112	230113
	230123	230133	230203	230213	230223	231000	231001	231002	231003	231010	231011	231012	231013
	231022	231032	231100	231101	231102	231103	231110	231111	231112	231113	231122	231132	231222
	232000	232001	232002	232003	232010	232011	232012	232013	232100	232101	232102	232103	232110
	232111	232112	232113	232201	232211	232221	232231	233001	233002	233003	233010	233011	233012
	233013	233100	233101	233102	233103	233111	233112	233113	233220	233230	300003	300013	300020
	300021	300022	300023	300030	300031	300032	300033	300103	300113	300120	300121	300122	300123
	300130	300131	300132	300133	300203	300213	301002	301012	301020	301021	301022	301023	301030
	301031	301032	301033	301102	301112	301120	301121	301122	301123	301130	301131	301132	301133
	301202	301212	301222	301232	302020	302021	302022	302023	302030	302032	302033	302120	302122
	302123	302130	302131	302132	302133	302201	302211	302301	303020	303021	303022	303023	303030
	303031	303032	303033	303120	303121	303122	303123	303130	303131	303132	303133	303200	303210
	303220	303230	303300	310003	310013	310020	310021	310022	310023	310030	310031	310032	310033
	310103	310113	310120	310121	310122	310123	310130	310131	310132	310133	310203	310213	310303
	311002	311012	311020	311021	311022	311023	311030	311031	311032	311033	311102	311112	311120
	311121	311122	311123	311130	311131	311132	311133	311202	311212	311222	311232	311302	312020
	312022	312023	312030	312031	312032	312033	312120	312121					

SUPPLEMENTARY REFERENCES

[1] T. Kriváchy, Y. Cai, D. Cavalcanti, A. Tavakoli, N. Gisin, and N. Brunner, "A neural network oracle for quantum nonlocality problems in networks," npj Quant. Info. **6**, 70 (2020).

[2] E. D. Andersen, "Certificates of Primal or Dual Infeasibility in Linear Programming," Comp. Optim. Appl. **20**, 171 (2001).
